# Impact of Oviposition Sequence and Host Egg Density on Offspring Emergence and Interspecific Competition in Two Species of *Trichogramma* Parasitoids

**DOI:** 10.3390/insects16020214

**Published:** 2025-02-15

**Authors:** Yu Wang, Asim Iqbal, Kanwer Shahzad Ahmed, Zheng-Kun Zhang, Juan Cui, Chen Zhang

**Affiliations:** 1 Agricultural College, Jilin Agricultural Science and Technology University, Jilin 132101, China; wangy19940504@163.com (Y.W.); cuijuanjilin@163.com (J.C.); 2 Imdaad: Integrated Facilities Management Company, Street Number 1100, South Zone Jebel Ali, Dubai PO Box 18220, United Arab Emirates; asim_iqbal990@yahoo.com; 3 Biological Research & Resource Center, Mastermind Scientific Consultants (SMC-Private) Limited, Sargodha 40100, Pakistan; rajput.shahzad18@gmail.com; 4 Institute of Plant Protection, Jilin Academy of Agricultural Science/Jilin Key Laboratory of Agricultural Microbiology/Key Laboratory of Integrated Pest Management on Crops in Northeast China, Ministry of Agriculture and Rural Areas, Gongzhuling 136100, China; zhangzhengkun1980@126.com

**Keywords:** Asian corn borer, biological control, emergence rate, rice moth, *Trichogramma ostriniae*, *Trichogramma dendrolimi*

## Abstract

Asian corn borer (ACB), *Ostrinia furnacalis* Guenee (Lepidoptera: Crambidae) and the rice moth (RM), *Corycyra cephalonica* Stainton (Lepidoptera: Pyralidae) are economically significant insect pests that threaten the agricultural products worldwide. *Trichogramma* parasitoids are successfully mass-reared using artificial host eggs, RM, and are often managed by economically important lepidopterous pests, such as ACB in China. *Trichogramma ostriniae* Pang and Chen (To) and *T. dendrolimi* Matsumura (Td) (Hymenoptera: Trichogrammatidae) are two important parasitoids of ACB. To determine the influence of interspecific competition between To and Td on their offspring’s emergence. We determine the emergence of wasp progeny from two distinct hosts (ACB and RM eggs) of varying densities (10, 20, 30, and 100) by examining the effect of three distinct oviposition sequences (To-Td, Td-To, and To+Td) by two *Trichogramma* species. We discovered that the progeny emergence rate of To and Td from the host was substantially influenced by the parasitoid types, host types, oviposition sequences, and host densities, and their two-, three-, and four-factor interactions while investigating the ACB and RM eggs after oviposition. Additionally, the progeny of Td emerged from 10, 20, and 30 ACB host eggs under three oviposition sequences, which was significantly higher than that of ACB eggs of 100 densities. Nevertheless, the optimum emergence rate of Td progeny was also observed in ACB eggs with a density of 100 under all oviposition sequences. The most suitable oviposition sequences for both wasp species are To-Td and Td-To, as they have the highest rate of progeny emergence. The progeny emergence of both *Trichogramma* species from RM eggs of varying densities was observed to be significantly different. Nevertheless, the most influential density is 100 RM eggs, as a result of the maximal emergence rate of To and Td. Overall, it is concluded that host eggs with a density of 100 are adequate to meet the oviposition requirements of both wasps in all oviposition orders, thereby limiting their interspecific competition. These findings provide insights into optimizing host density and oviposition strategies for mass-rearing *Trichogramma* species, which can enhance their efficacy in biological control programs. Future research should explore field-level applications to validate these laboratory findings under natural conditions.

## Simple Summary

For assessing interspecific competition, the offspring emergence of two egg parasitoids, *Trichogramma ostriniae* Pang and Chen (To) and *T. dendrolimi* Matsumura (Td), were compared on two different host eggs (Asian corn borer (ACB), Ostrinia furnacalis Guenee and rice moth (RM), *Corcyra cephalonica* Stainton) of varying densities (10, 20, 30 and 100) under three distinct oviposition sequences (To-Td, Td-To, and To+Td). The results indicated that the progeny emergence rate of To and Td from the host was substantially influenced by the parasitoid types, host types, oviposition sequences, and host densities, and their two-, three-, and four-factor interactions while investigating the ACB and RM eggs after oviposition. The most suitable oviposition sequences for both wasp species are To-Td and Td-To, as they have the highest rate of progeny emergence. Overall, it is concluded that host eggs with a density of 100 are adequate to meet the oviposition requirements of both wasps in all oviposition orders.

## 1. Introduction

The Asian corn borer (ACB), *Ostrinia furnacalis* Guenee (Lepidoptera: Crambidae), is an important agricultural pest that was first identified in 1854 and widely distributed throughout the Asia Pacific regions, including China, Japan, and Australia [1,2,3,4,5,6]. In China, maize (*Zea mays*, Linnaeus) is the main host plant of ACB, but it is also found on a range of other plants, including rice (*Oryza sativa* Linnaeus), sorghum (*Sorghum bicolor* (Linnaeus) Moench), and soybean (*Glycine max* (Linnaeus) Merr.) [7]. While infesting corn, the ACB larvae consumed leaves, stalks, and tassels and caused severe yield loss [8]. Like ACB, the rice moth (RM), *Corycyra cephalonica* Stainton (Lepidoptera: Pyralidae)*,* is another economically significant insect pest. It threatens agricultural stored products on a global scale, resulting in substantial losses for cereals, grain legumes, and other high-value commodities, including dried fruits and cacao beans [9,10,11]. Currently, chemical insecticides are the primary method of prevention and control for ACB and RM. Nevertheless, the improper application of chemical insecticides can elevate insect pest resistance levels [12]. Additionally, the application of insecticides is highly undesirable for effective insect control due to the high cost, environmental pollution, and health risks to farmers [13]. Consequently, it is essential to establish a sustainable management of ACB and RM that employs natural enemies in response to the detrimental effects of synthetic insecticides.

The *Trichogramma* wasps are considered among the most important parasitoid natural enemies of insect pests worldwide. China has historically employed *Trichogramma* (Hymenoptera: Trichogrammatidae) to effectively manage lepidopteran pests, such as cotton (*Gossypium arboretum* Linnaeus), maize, rice, sorghum, soybean, sugar cane (*Saccharum officinarum* Linnaeus), and tomato (*Solanum lycopersicum* Linnaeus) [14]. Additionally, the utilization of *Trichogramma* is highly industrialized in China [14]. From 2005 to 2015, the area of maize in Northeastern China that relied on *Trichogramma* releases for corn borer control increased from 600,000 to 5,500,000 ha, accounting for thirty-five percent of the area under corn cultivation in China’s most important corn production region [15,16]. Trichogrammatidae comprises approximately 620 species, with 219 species recorded in China [17,18]. Wang et al. [19] have reported that *Trichogramma* has been subjected to substantial mass cultivation on RM since the 1970s and has been disseminated on a large scale in China to regulate ACB. *Trichogramma ostriniae* Pang and Chen and *T. dendrolimi* Matsumura are the primary egg parasitoid species that are employed as key tools in biological control programs [14].

Suppressing the insect pest population is essential for the biological control program. One of the most challenging concerns in biological control has been whether a single parasitoid substantially reduces the abundance of the insect pest’s population compared to a combination of parasitoids due to the effect of competition for shared resources [20]. For instance, Cusumano et al. [21] asserted that parasitoid species engage in interspecific competition for resources during their adult and larval stages, impacting the parasitoid’s fitness. He et al. [22] examined the interspecific competition between *Trichogramma confusum* Jacquelin du Val and *T. pretiosum* Riley on the rice moth, *Corcyra cephalonica* Stainton, under a variety of parasitoid densities and host densities. The results suggested that the parasitism increased with parasitoid density in single and mixed inoculated groups, while it decreased with host density. Additionally, in mixed inoculated groups, the proportion of *T. pretiosum* progeny decreased with parasitoid density but increased with host density. This indicates that *T. pretiosum* possessed a more robust competitive ability than *T. confusum*, most likely due to the increased number of odorant binding proteins and odorant receptors in olfactory tissue (antennae) [23]. Furthermore, for a biological control program to be successful, it is essential to monitor the capacity of parasitoids to parasitize hosts at varying densities [24,25]. The behavioral responses of *Trichogramma* species to host variations are the prospective indicators of quality and field success [26]. Consequently, it is imperative to identify the *Trichogramma* species most effective in developing target pest eggs and evaluate a variety of host densities before releasing them into the field [27].

The oviposition sequence substantially influences the efficacy of parasitoid progeny during interspecific competition. For instance, Luo et al. [28] examined the oviposition sequence of two single nymphal parasitoids (*Peristenus spretus* Chen & van Achterberg and *P. relictus* Loan) of the green plant insect *Apophylgus lucorum* Meyer-Durin to assess nutrient competition between the two species. The scientists permitted a female of each parasitoid species to sting a nymph of a green plant insect only once. Two parasitism sequences, Ps-Pr and Pr-Ps, were implemented. In the Ps-Pr sequence, a single female *P. spretus* parasitized a single host nymph, which was subsequently offered to *P. relictus*. One nymph was parasitized by a single *P. spretus* female in the Pr-Ps sequence following *P. relictus*. The results indicated that in every sequence, *P. spretus* was the dominant adult parasitoid that emerged in the progeny generation. This is because *P. relictus* immature stages lasted 2.2 days longer than *P. spretus* in the host body. As a result, the progeny of *P. spretus* emerged earlier than those of *P. relictus* and initiated intrinsic competition by biting and physically attacking the immature *P. relictus* with sickle-shaped mandibles [28]. As a result, the dominant parasitoid induces physiological stress on competing parasitoids and monopolizes host resources [29]. The oviposition sequence plays a pivotal role in determining the outcome of interspecific competition among parasitoid species by influencing progeny development timing and survival. Thus, the purpose of this investigation was to evaluate the results of multiparasitism when a range of densities of hosts (ACB and RM eggs) was sequentially exposed to the two parasitoids (*T. ostriniae* and *T. dendrolimi*), with the same time interval between the first and second parasitoid species. The research findings establish a theoretical foundation for the further scientific and rational implementation of *Trichogramma* in the management of ACB and RM.

## 2. Materials and Methods

### 2.1. Parasitoids

In 2011, adult *T. ostriniae* (To) and *T. dendrolimi* (Td) were obtained from parasitized ACB eggs in the cornfields of Changchun, Jilin Province, China. Both parasitoid species were identified based on the morphological characteristics of the male genitalia using electronic microscope micrographs [30], and rDNA-ITS2 sequence analysis confirmed this identification [31]. To maintain their initial parasitization capacity, these parasitoids were cultured on RM eggs for five generations in an incubator (MLR-351H; Sanyo Corporation, Osaka, Japan) under feasible conditions (L14: D10, 26 ± 1 °C, 65 ± 5% RH). Td and To colonies were subsequently maintained on their native host (ACB eggs) for a single generation.

### 2.2. Hosts

#### 2.2.1. Asian Corn Borer (ACB), *Ostrinia furnacalis*

ACB pupae were maintained in a cage (35 × 35 × 35 cm; Bugdorm-I, Taiwan, China) at optimal conditions (L14:D10, 26 ± 1 °C, 65 ± 5% RH) to collect eggs for experimentation. Following their emergence, the moths were provided with a 10% honey solution as a sustainable food source, and strips of wax paper were suspended for oviposition. After oviposition, the egg masses were cut with a scissor and deposited in a climate chamber room (Faithful Instrument Co., Ltd., Huanghua, Hebei, China) under optimal conditions (L14:D10, 26 ± 1 °C, 65 ± 5% RH) until they attained the appropriate age for experimentation.

#### 2.2.2. Rice Moth (RM), *Corcyra cephalonica*

For egg collection, the RM larvae were maintained in a plastic container and provided with a corn-based artificial diet until pupation [32]. The container’s sanitation was maintained consistently. The emerged RM adults were collected and placed into mesh cages (35 cm× 35 cm× 35 cm) for oviposition, which were then placed on the egg-collecting tray. The oviposition was periodically checked, and the eggs laid by female RM on the cage walls were swept into the tray using a collecting brush. Furthermore, the collected RM eggs were filtered using a 0.5 mm net to eliminate dirt and scales, resulting in clean eggs. The eggs were stored in a climate chamber room at ideal circumstances (L14:D10, 26 ± 1 °C, 65 ± 5% RH) until they reached the required age for testing.

### 2.3. Interspecific Competition Between Two Trichogramma Species Within the ACB and RM Eggs

In order to assess the outcome of interspecific competition between To and Td, a female of each parasitoid species was allowed to parasitize ACB eggs of varying densities in a glass tube (3 cm in diameter and 10 cm in length; Zhejiang Runlab Technology Co., Ltd., Taizhou, Zhejiang, China) for same time interval under controlled conditions (L14:D10, 26 ± 1 °C, 65 ± 5% RH). Three parasitism sequences (species order: To-Td, Td-To, and To+Td) were employed in this experiment, with a variation in the host (ACB eggs: 10, 20, 30, and 100). The To-Td sequence involved placing ACB eggs (0–4 h old) of varying densities in different glass tubes (3 cm in diameter and 10 cm in length). Subsequently, a single To female (newly emerged and mated adult) was introduced into each glass tube for parasitization for four hours. After initial oviposition, the To was removed, and then a single Td female (newly emerged and mated adult) was dropped in the same test tubes for multiparasitism for the same time interval. In the Td-To sequence, the same procedures with the same set-up of host densities were applied. The To+Td sequence included placing ACB eggs (0–4 h old) of varying densities in various glass tubes (3 cm in diameter and 10 cm in length). Then, a single To and a single Td female (newly emerged and mated adults) were introduced simultaneously into each glass tube for a four-hour parasitization period. The parasitized egg masses were transferred to an incubator for development under controlled conditions (L14:D10, 26 ± 1 °C, 65 ± 5% RH) following parasitism. The progression of parasitoid offspring emergence was regularly monitored until it was complete. Each treatment was replicated fifteen times. The same experimental procedure, including parasitism sequences, host egg densities, and controlled conditions, was applied to evaluate the interspecific competition between To and Td using RM eggs.

### 2.4. Statistical Analysis

Data were analyzed using the statistical software IBM SPSS^®^ (Statistical Package for Social Science, IBM, New York, and United States) v8.0. The data regarding offspring emergence rate was analyzed using factorial analysis of variance (ANOVA), and treatment means were further compared using Tukey’s honestly significant difference (HSD) test at a 95% confidence interval. Data were displayed using SigmaPlot 12.5.

## 3. Results

The regression analysis showed that the offspring emergence rate of *Trichogramma* species was significantly affected by parasitoid types (*p* = 0.000), host types (*p* = 0.005), oviposition sequences (*p* = 0.041), host densities (*p* = 0.016), parasitoid types × host types (*p* = 0.000), parasitoid types × oviposition sequences (*p* = 0.000), parasitoid types × host densities (*p* = 0.000), host types × oviposition sequences (*p* = 0.043), host types × host densities (*p* = 0.040)

oviposition sequences × host densities (*p* = 0.040), parasitoid types × host types × oviposition sequences (*p* = 0.000), parasitoid types × host types × host densities (*p* = 0.000),

parasitoid types × oviposition sequences × host densities (*p* = 0.000), host types × oviposition sequences × host densities (*p* = 0.040), and parasitoid types × host types × oviposition sequences × Host densities (*p* = 0.000) (Supplementary Table S1).

### 3.1. Influence of Wasp Oviposition Sequence and Host Densities on Offspring Emergence of Trichogramma Parasitoids from ACB Eggs

**Figure 1:**
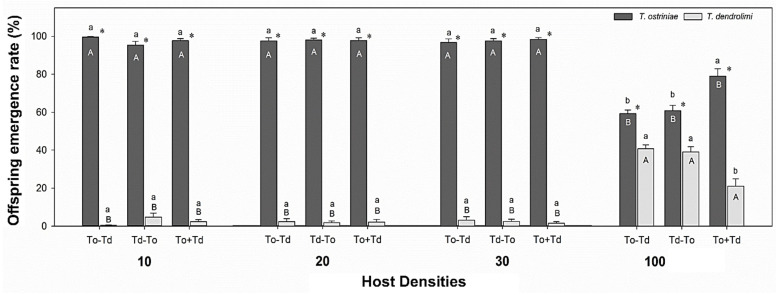
Percentage (mean ± SE) of emerged offspring of *Trichogramma ostriniae* (To) and *Trichogramma dendrolimi* (Td) from *Ostrinia furnacalis* host of different densities after various oviposition sequences. To-Td represents the initial introduction of To for four hours, and Td is introduced for the same time interval after its removal; Td-To indicates the initial introduction of Td for four hours, while To is introduced for the same time interval after its removal. To+Td denotes the simultaneous introduction of To and Td for four hours. Different upper-case letters on the same patterned bars indicate significant statistical differences in the offspring emergence rate of To and Td from different numbers of *O. furnacalis* eggs, while different lower-case letters on the bars within a given group indicate significant statistical differences in the offspring emergence rate of To and Td from the same number of *O. furnacalis* eggs (Tukey’s HSD test, *p* < 0.05). The paired bars with asterisk indicate significant differences in means (*p* < 0.05).

Under the same oviposition sequence, we compared the effects of ACB densities on the offspring emergence of To and Td. We found significant differences in the offspring emergence of Td among different host egg densities [Td (To-Td): F3,53 = 100.153, *p* < 0.0001, Td (Td-To): F3,54 = 40.416, *p* < 0.0001, and Td (Td+To): F3,53 = 18.167, *p* < 0.0001] (Figure 1). Under three oviposition sequences, the Td progeny emergence from 10 [Td (To-Td): 0.34%, Td (Td-To): 4.68%, Td (Td+To): 2.32%], 20 [Td (To-Td): 2.34%, Td (Td-To): 1.87%, Td (Td+To): 2.16%] and 30 [Td (To-Td): 3.18%, Td-To: 2.46%, Td+To: 1.51%] ACB host eggs was significantly lower than ACB eggs of 100 densities [Td (To-Td): 40.79%, Td (To-Td): 39.10%, Td (Td+To): 20.94%] (Figure 1).

Furthermore, we also found significant differences in the offspring emergence of To among different host egg densities [To (To-Td): F3,53 = 100.153, *p* < 0.0001, To (Td-To): F3,54 = 40.416, *p* < 0.0001, To (Td+To): F3,53 = 18.167, *p* < 0.0001] (Figure 1). Under three oviposition sequences, the offspring of To emerged from 10 [To (To-Td): 99.66%, To (Td-To): 95.32%, To (Td+To): 97.68%], 20 [To (To-Td): 97.66%, To (Td-To): 98.12%, To (Td+To): 97.84%], and 30 [To (To-Td): 96.82%, To (Td-To): 97.54%, To (Td+To): 98.49%] ACB host eggs, were significantly higher than that emerged from ACB eggs of 100 densities [To (To-Td): 59.21%, To (Td-To): 60.90%, To (Td+To): 79.06%] (Figure 1).

We compared the effects of varying host densities on the progeny emergence rate of Td. Our findings indicate that the emergence of Td is not significantly different among various sequences of oviposition on 10, 20, and 30 eggs of ACB (10 eggs Td: F2,39 = 2.519, *p* = 0.0936; 20 eggs Td: F2,40 = 0.020, *p* = 0.9798; 30 eggs Td: F2,42 = 0.237, *p* = 0.7897) (Figure 1). Nevertheless, a substantial difference was observed when 100 eggs of ACB were supplied (100 eggs Td: F2,42 = 9.242, *p* = 0.0005). Among them, there were no substantial variations in the percentage of emerged Td offspring between the (To-Td) and (Td-To) sequences; however, both oviposition sequences substantially emerged more Td than those from the To+Td sequence (Figure 1).

Additionally, the To emergence rate on 10, 20, and 30 eggs of ACB did not exhibit a significant difference among the various orders of oviposition [10 eggs (To): F2,39 = 2.519, *p* = 0.0936, 20 eggs (To): F2,40 = 0.020, *p* = 0.9798, 30 eggs (To): F2,42 = 0.237, *p* = 0.7897]. However, a significant difference was observed on 100 eggs of ACB (100 eggs To: F2,42 = 9.242, *p* = 0.0005). The progeny emergence rate of To under the (To-Td) and (Td-To) sequences was not significantly different; however, both oviposition sequences substantially emerged lower To than those from the To+Td sequence. In summary, the results indicate that the progeny emergence rates of Td and To differed significantly under the same oviposition sequence and number of host eggs. Td was significantly lower than To in all treatments (All *p* < 0.05) (Figure 1).

### 3.2. Influence of Wasp Oviposition Sequence and Host Densities on Offspring Emergence of Trichogramma Parasitoids from RM Eggs

**Figure 2:**
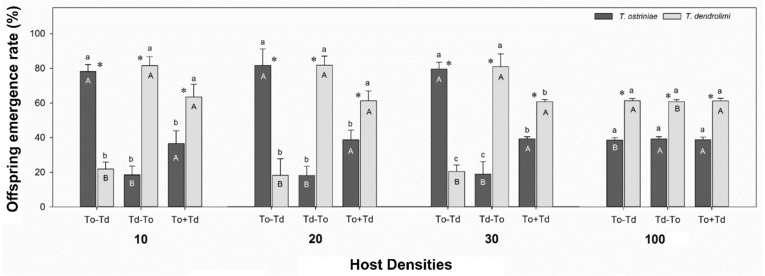
Percentage (mean ± SE) of emerged offspring of *Trichogramma ostriniae* (To) and *Trichogramma dendrolimi* (Td) from *Corcyra cephalonica* host of different densities after various oviposition sequence. To-Td represents the initial introduction of To for four hours, and Td is introduced for the same time interval after its removal; Td-To indicates the initial introduction of Td for four hours, while To is introduced for the same time interval after its removal. To+Td denotes the simultaneous introduction of To and Td for four hours. Different upper-case letters on the same patterned bars indicate significant statistical differences in the offspring emergence rate of To and Td from different numbers of *C. cephalonica* eggs, while different lower-case letters on the bars within a given group indicate significant statistical differences in the offspring emergence rate of To and Td from the same number of *C. cephalonica* eggs (Tukey’s HSD test, *p* < 0.05). The paired bars with asterisk indicate significant differences in means (*p* < 0.05).

We found significant differences in the offspring emergence of Td among different host egg densities under two oviposition sequences [Td (To-Td): F3,54 = 14.822, *p* < 0.0001, Td (Td-To): F3,53 = 4.069, *p* = 0.0113] (Figure 2). The To-Td sequence did not reveal any significant differences in the progeny emergence rate of Td from 10 (21.87%), 20 (18.29%), and 30 (20.40%) eggs of RM. Nevertheless, a significantly higher percentage (61.36%) of Td emergence was observed from 100 RM eggs. Conversely, the Td-To sequence demonstrated a substantially reduced incidence of Td emergence from 100 RM eggs (60.73%) than from 10, 20, and 30 RM eggs (81.59%, 81.81%, and 81.06%, respectively). No significant differences were observed in the emerged Td from 10 (63.43%), 20 (61.27%), 30 (61.15%), and 100 (61.18%) RM eggs concerning the Td+To sequence [Td (Td+To): F3,55 = 0.054, *p* = 0.9835] (Figure 2).

Regarding the comparison of the emergence of To offspring from various oviposition sequences, we observed substantial variations in the emergence of To offspring among varying host egg densities under two oviposition sequences [To (To-Td): F3,54 = 14.822, *p* < 0.0001, To (Td-To): F3,53 = 4.069, *p* = 0.0113]. (See Figure 2). The progeny emergence rate of To from 10 (78.13%), 20 (81.71%), and 30 (79.60%) eggs of RM did not exhibit any significant differences in the To-Td sequence (Figure 2). However, a considerably reduced percentage (38.64%) of To emergence was observed in 100 RM eggs. In contrast, the Td-To sequence exhibited a significantly higher rate of To emergence from 100 RM eggs (39%) than from 10, 20, and 30 RM eggs (19.54%, 19.10%, and 20.10%, respectively). The emerged To from 10 (36.57%), 20 (38.73%), 30 (38.85%), and 100 (38.82%) RM eggs did not exhibit any significant differences in the Td+To sequence [Td (Td+To): F3,55 = 0.054, *p* = 0.9835] (Figure 2).

The effects of various oviposition sequences on the emergence rate of Td and To adult wasps were compared under the same number of host eggs. We found that the highest percentage of Td emergence from the density of 10 RM eggs was observed under the Td-To (78.13%) and the lowest under the To-Td (21.87%). In contrast, the To progeny produced under the Td-To oviposition sequence exhibited a reduced emergence rate (18.54%), while the To-Td sequence exhibited a higher emergence rate (78.13%). The RM host exhibited the same trend of Td and To emergence at densities of 20 and 30, respectively. Moreover, the same emergence rate of Td and To progeny was observed among To-Td, Td-To, and To+Td oviposition sequences (Figure 2). In conclusion, the findings suggest that the progeny emergence rates of Td and To varied considerably under the same oviposition sequence and number of RM eggs (Figure 2).

## 4. Discussion

The density and availability of host resources are crucial factors in the reproduction of parasitoid wasp populations [33,34]. When parasitoid wasps encounter a scarcity of host resources, they typically engage in intense competition with one another [35]. This study demonstrated the impact of host density and various oviposition sequences on the emergence of To and Td progeny from ACB and RM eggs. However, an in-depth comparison between these two host types reveals critical differences that influence competitive outcomes and parasitoid performance. Our findings suggest that ACB eggs are more favorable for To, whereas Td exhibits relatively better performance on RM eggs. The higher emergence rate of To from ACB eggs across different densities and oviposition sequences suggests that this host is more compatible with To’s reproductive strategy. The presence of kairomones (*E*)-12-tetradecenyl acetate in ACB eggs enhances host-finding efficiency for To [36], likely giving it a competitive edge in this environment. Conversely, Td emergence was more consistent in RM eggs, suggesting a stronger host-parasitoid relationship. Our results align with those of Liu et al. [37], who revealed that RM eggs are the most preferred hosts over ACB eggs by Td. Furthermore, *Trichogramma chilonis* Ishii also used kairomone, (*Z*)-11-hexadecenyl acetate for finding *Helicoverpa assulta* Guenee eggs [38]. Therefore, kairomones secreted by host eggs indicated the suitability of the host for parasitoid progeny.

Host density significantly impacted the competitive dynamics between To and Td across both host types. In ACB eggs, To emergence was consistently higher than Td, particularly at lower densities (10, 20, 30 eggs), where competition for resources was more pronounced. This suggests that To, which has a faster development rate, outcompeted Td when resources were limited [39]. However, when ACB egg density was increased to 100, Td emergence improved, indicating that sufficient resources can mitigate interspecific competition. For the RM eggs, the competitive trend was different. The emergence of Td was consistently high across all densities, particularly in the To+Td oviposition sequence, where both parasitoids were introduced simultaneously. This suggests that Td is better adapted to RM eggs, possibly due to its evolutionary history of mass rearing on RM as a factitious host [19,40]. Unlike ACB eggs, where To showed a dominant emergence pattern, both species exhibited relatively balanced emergence in RM eggs, indicating a less aggressive competitive interaction.

The oviposition sequence played a crucial role in determining the emergence success of To and Td, although its impact varied depending on the host type. In ACB eggs, the To-Td and Td-To sequences led to significantly higher emergence rates for To, reinforcing its superior ability to exploit its host [39,41]. The To+Td sequence, where both species oviposited simultaneously, still favored To but showed a relative increase in Td emergence at higher host densities, suggesting that competition was moderated when resource availability was ample. For RM eggs, the effect of the oviposition sequence was less pronounced. While variations in emergence rates were observed in the To-Td and Td-To sequences, the To+Td sequence led to comparable emergence rates for both species. This finding suggests that Td is capable of competing more effectively in RM eggs than in ACB eggs, further supporting the premise that RM serves as a more suitable host for Td. In China, RM eggs are frequently employed as an effective intermediate host for the mass propagation of *Trichogramma* [19,40]. Td is also extensively mass-reared on RM to manage lepidopteran parasites in China [42], and consequently, it is more compatible with the RM host egg than other wasp species. Td was also found to have superior temperature and humidity adaptation to other *Trichogramma* species by Tang, Sun [43]. Consequently, it is a promising candidate for controlling insect pests in various environmental conditions.

The observed host-dependent differences in parasitoid emergence have significant implications for biological control programs. The To is superior to Td in controlling ACB because of higher offspring emergence rates and a competitive advantage. Conversely, RM eggs appear to be a more stable and suitable host for Td, allowing for consistent emergence without significant competitive suppression by To. These findings underscore the necessity of selecting appropriate host species when rearing and deploying *Trichogramma* parasitoids for pest management. Future research should extend these laboratory-based insights to field conditions, where additional ecological variables may further influence parasitoid competition and host preference. A comprehensive understanding of parasitoid interactions, host suitability, and oviposition dynamics will contribute to optimizing the efficacy of biological control programs targeting lepidopterans pests.

## 5. Conclusions

According to the findings of the study as a whole, the emergence of To and Td progeny is influenced by the density of the host egg and the oviposition sequences. Under lower host density, the wasps’ competition for shared resources ultimately compromises the emergence of progeny. Host density is more critical than oviposition sequences to optimize the offspring emergence rate of both wasp species. Therefore, to mitigate interspecific competition among offspring, the maximal host density of ACB and RM eggs was implemented during selected oviposition sequences. However, the future perspective of this investigation is to evaluate this influence in field conditions.

## Data Availability

The raw data supporting the conclusions of this article will be made available by the authors upon request.

## Supplementary Materials

The following supporting information can be downloaded at: https://www.mdpi.com/article/10.3390/insects16020214/s1, Table S1: Four-way ANOVA results indicating the effect of parasitoid types, host types, oviposition sequences and host densities on offspring emergence rate of *Trichogramma* species.

## References

[1] Guo J., He K., Meng Y., Hellmich R.L., Chen S., Lopez M.D., Lauter N., Wang Z. (2022). Asian corn borer damage is affected by rind penetration strength of corn stalks in a spatiotemporally dependent manner. Plant Direct..

[2] Yuan Z.G., Guo J.F., Wang Z., He K., Bai S. (2013). Feeding preference of the Asia corn borer larvae for different host plants. Acta Phytophy. Sin.

[3] Yuan Z.H., Wang W.Q., Wang Z.Y., He K.L., Bai S.X. (2015). Host plants of the Asian corn borer, *Ostrinia furnacalis* (Guenee) (Lepidoptera: Crambidae). J. Plant Prot..

[4] Afidchao M.M., Musters C.J., de Snoo G.R. (2013). Asian corn borer (ACB) and non-ACB pests in GM corn (*Zea mays L*.) in the Philippines. Pest. Manag. Sci..

[5] Rahayu T., Trisyono Y.A., Witjaksono (2018). Fitness of Asian corn borer, *Ostrinia furnacalis* (Lepidoptera: Crambidae) reared in an artificial diet. J. Asia Pac. Entomol..

[6] Chen R.Z., Klein M.G., Li Q.Y., Li L.B., Li P.P., Sheng C.F. (2015). Do second generation Asia corn borer (Lepidoptera: Crambidae) immigrate to corn fields from alternate habitats?. J. Asia Pac. Entomol..

[7] Wei X., Chen R.Z. (2020). Effects of host plants on the development and protective enzyme activity of Ostrinia furnacalis. Chin. J. Appl. Entomol..

[8] Nafus D.M., Schreiner I.H. (1991). Review of the biology and control of the Asian corn borer, *Ostrinia furnacalis* (Lep: Pyralidae). Trop. Pest. Manag..

[9] Vincent A., Singh D., Mathew I.L. (2021). *Corcyra cephalonica*: A serious pest of stored products or a factitious host of biocontrol agents?. J. Store. Prod. Res..

[10] Adarkwah C., Nyarko G., Opoku N., Badii B.K., Addai I.K., Prozell S., Ulrichs C., Schöller M. (2015). Effectiveness of the egg parasitoid *Trichogramma evanescens* preventing rice moth from infesting stored bagged commodities. J. Store. Prod. Res..

[11] Manjunath T.M. (2023). Rice moth, *Corcyra cephalonica* (Lepidoptera, Pyralidae)—A boon for biocontrol as a factitious host for mass production of parasitoids and predators. J. Biol. Cont..

[12] Sun C., Li S., Wang K., Yin X., Wang Y., Du M., Wei J., An S. (2022). Cyclosporin A as a potential insecticide to control the Asian corn borer Ostrinia furnacalis Guenée (Lepidoptera: Pyralidae). Insects.

[13] Liu M., Hao Y., Sun Y., Wang J. (2016). Developing insect resistance with fusion gene transformation of chitinase and scorpion toxin gene in maize (*Zea mays* L). Maydica.

[14] Zang L.S., Wang S., Zhang F., Desneux N. (2021). Biological control with *Trichogramma* in China: History, present status, and perspectives. Ann. Rev. Entomol..

[15] Zhang J., Ruan C., Zang L., Shao X., Shi S. (2015). Technological improvements for mass production of *Trichogramma* and current status of their applications for biological control on agricultural pests in China. Chi. J. Biol. Cont..

[16] Huang N.X., Jaworski C.C., Desneux N., Zhang F., Yang P.Y., Wang S. (2020). Long-term and large-scale releases of *Trichogramma* promote pesticide decrease in maize in northeastern China. Entomol. Gen..

[17] Pinto J.H., Stouthamer R. (1994). Systematics of the Trichogrammatidae with emphasis on Trichogramma. Biological Control with Egg Parasitoids.

[18] Vinson S.B., Greenberg S.M., Rao A., Volosciuk L.F. (2015). Biological Control of Pests Using Trichogramma: Current Status and Perspectives.

[19] Wang Z.Y., He K.L., Zhang F., Lu X., Babendreier D. (2014). Mass rearing and release of *Trichogramma* for biological control of insect pests of corn in China. Biol. Cont..

[20] Mills N., Brodeur J., Boivin G. (2006). Interspecific competition among natural enemies and single versus multiple introductions in biological control. Trophic and Guild in Biological Interactions Control.

[21] Cusumano A., Peri E., Colazza S. (2016). Interspecific competition/facilitation among insect parasitoids. Curr. Opin. Insect Sci..

[22] He Y., Lü L., Chen K. (2004). Interspecific competition between *Trichogramma confusum* and *T. pretiosum* on *Corcyra* cephaloica factitious eggs. Chin. J. Appl. Ecol..

[23] Al-Jalely B.H., Xu W. (2021). Olfactory sensilla and olfactory genes in the parasitoid wasp *Trichogramma pretiosum* Riley (Hymenoptera: Trichogrammatidae). Insects.

[24] Lin L., Ali S., Wu J. (2018). Influences of varying host: Parasitoid ratios on parasitism of whitefly by three different parasitoid species. Egypt. J. Biol. Pest. Cont..

[25] Saljoqi A.U., Salim M., Khalil S.K., Khurshid I. (2015). Field application of *Trichogramma chilonis* (Ishii) for the management of sugarcane borers. Pak. J. Zool..

[26] Bigler F., Cerutti F., Laing J. (1991). First draft of criteria for quality control (product control) of *Trichogramma*. Fifth Workshop of the IOBC Global Working Group on Quality Control of Mass Reared Arthropods.

[27] Sani I.A., Shabbir S., Zafar M., Ahmed N., Shahwani N.A., Yousafzai A., Irfan S., Ahmed U., Aziz S., Khan A.G. (2016). Biological control of insect pests using *Trichogramma minutum* as biological control agent in the vicinity of buitems. COMU J. Agric. Fac..

[28] Luo S., Zhang F., Wu K. (2018). Interspecific competition between *Peristenus spretus* and *Peristenus relictus* (Hymenoptera: Braconidae), larval parasitoids of *Apolygus lucorum* (Hemiptera: Miridae). Biol. Cont..

[29] Harvey J.A., Poelman E.H., Tanaka T. (2013). Intrinsic inter- and intraspecific competition in parasitoid wasps. Annu. Rev. Entomol..

[30] Pinto J.D. (1992). Novel taxa of *Trichogramma* from the New World Tropics and Australia (Hymenoptera: Trichogrammatidae). J. N. Y. Entomol. Soc..

[31] Stouthamer R., Hu J., van Kan F.J., Platner G.R., Pinto J.D. (1999). The utility of internally transcribed spacer 2 DNA sequences of the nuclear ribosomal gene for distinguishing sibling species of *Trichogramma*. BioControl.

[32] Pinto J.R., Torres A.F., Truzi C.C., Vieira N.F., Vacari A.M., De Bortoli S.A. (2019). Artificial corn-based diet for rearing *Spodoptera frugiperda* (Lepidoptera: Noctuidae). J. Insect Sci..

[33] Bolter C.J., Laing L.E. (1983). Competition between *Diadegma insulare* (Hymenoptera: Ichneumonidae) and *Microplitis plutellae* (Hymenoptera: Braconidae) for larvae of the diamondback moth, *Plutella xylostella* (Lepidoptera: Plutellidae). Proc. Entomol. Soc. Ont..

[34] Hawkins B.A. (1988). Species diversity in the third and fourth trophic levels: Patterns and mechanisms. J. Ani. Ecol..

[35] Hågvar E.B. (1989). Interspecific competition in parasitoids, with implications for biological control. Acta Entomol. Bohemoslov..

[36] Bai S., Wang Z., He K., Wen L., Zhou D. (2004). Olfactory responses of *Trichogramma ostriniae* Pang et Chen to kairomones from eggs and different stages of female adults of *Ostrinia furnacalis* (Guenee). Kun Chong Xue Bao Acta Entomol. Sin..

[37] Liu S.S., Zhang G.M., Zhang F. (1998). Factors influencing parasitism of *Trichogramma dendrolimi* on eggs of the Asian corn borer, *Ostrinia furnacalis*. BioControl.

[38] Boo K.S., Yang J.P. (2000). Kairomones used by *Trichogramma chilonis* to find *Helicoverpa assulta* eggs. J. Chem. Ecol..

[39] Wang Y., Hou Y.Y., Benelli G., Desneux N., Ali A., Zang L.S. (2022). *Trichogramma ostriniae* is more effective than *Trichogramma dendrolimi* as a biocontrol agent of the Asian corn borer, *Ostrinia furnacalis*. Insects.

[40] Subandi M., Setiati Y., Mutmainah N.H. (2017). Suitability of *Corcyra cephalonica* eggs parasitized with *Trichogramma japonicum* as intermediate host against sugarcane borer *Chilo auricilius*. Bulg. J. Agric. Sci..

[41] Myint Y.Y., Bai S., Zhang T., Babendreier D., He K., Wang Z. (2022). Ovipositional preference of *Trichogramma dedrolimi* and *Trichogramma ostriniae* strains from Myanmar on different host egg ages of Asian corn borer, *Otrinia furnacalis* (Lepidoptera: Crambidae). Biocont. Sci. Technol..

[42] Qu Y., Chen X., Monticelli L.S., Zhang F., Desneux N., Huijie D., Ramirez-Romero R., Wang S. (2020). Parasitism performance of the parasitoid *Trichogramma dendrolimi* on the plum fruit moth *Grapholitha funebrana*. Entomol. Gen..

[43] Tang L.D., Sun J.W., Dai P., Mu M.Y., Nkunika P.O., Desneux N., Zang L.S. (2023). Performance of two dominant trichogrammatid species of fall armyworm from China and Africa under contrasted temperature and humidity regimes. Biol. Cont..

