# Supplementary material for: Impact of Oviposition Sequence and Host Egg Density on Offspring Emergence and Interspecific Competition in Two Species of Trichogramma Parasitoids
Source: Insects. 2025 Feb 15;16(2):214. doi: 10.3390/insects16020214 (PMC11856026; doi:10.3390/insects16020214)
Supplement: Supplementary file 1 [file insects-16-00214-s001.zip › insects-3417386-supplementary.pdf]

Supplementary Table S1. Four-way ANOVA results indicating the effect of parasitoid types, host types, oviposition sequences and host densities on offspring emergence rate of *Trichogramma* species.

Parameter	Factor	df	F-value	P- value
Offspring emergence rate (%)	Parasitoid types	1	937.931	0.000
	Host types	1	7.762	0.005
	Oviposition sequences	2	2.987	0.041
	Host densities	3	3.459	0.016
	Parasitoid types × Host types	1	1523.226	0.000
	Parasitoid types × Oviposition sequences	2	130.712	0.000
	Parasitoid types × Host densities	3	78.127	0.000
	Host types × Oviposition sequences	2	3.161	0.043
	Host types × Host densities	3	1.492	0.040
	Oviposition sequences × Host densities	6	1.411	0.040
	Parasitoid types × Host types × Oviposition sequences	2	189.023	0.000
	Parasitoid types × Host types × Host densities	3	18.619	0.000
	Parasitoid types × Oviposition sequences × Host densities	6	21.372	0.000
	Host types × Oviposition sequences × Host densities	6	2.687	0.040

Parasitoid types × Host types × Oviposition sequences × Host densities	6	18.066	0.000
Error	665		

$p < 0.05$ is considered significant.
